# The male germline of angiosperms: repertoire of an inconspicuous but important cell lineage

**DOI:** 10.3389/fpls.2015.00173

**Published:** 2015-03-20

**Authors:** Scott D. Russell, Daniel S. Jones

**Affiliations:** Department of Microbiology and Plant Biology, University of OklahomaNorman, OK, USA

**Keywords:** angiosperm sperm cells, male chromatin modification, male gamete expression, male germ lineage, pollen

## Abstract

The male germline of flowering plants constitutes a specialized lineage of diminutive cells initiated by an asymmetric division of the initial microspore cell that sequesters the generative cell from the pollen vegetative cell. The generative cell subsequently divides to form the two male gametes (non-motile sperm cells) that fuse with the two female gametophyte target cells (egg and central cells) to form the zygote and endosperm. Although these male gametes can be as little as 1/800th of the volume of their female counterpart, they encode a highly distinctive and rich transcriptome, translate proteins, and display a novel suite of gamete-distinctive control elements that create a unique chromatin environment in the male lineage. Sperm-expressed transcripts also include a high proportion of transposable element-related sequences that may be targets of non-coding RNA including miRNA and silencing elements from peripheral cells. The number of sperm-encoded transcripts is somewhat fewer than the number present in the egg cell, but are remarkably distinct compared to other cell types according to principal component and other analyses. The molecular role of the male germ lineage cells is just beginning to be understood and appears more complex than originally anticipated.

The male gametophyte (pollen) of angiosperms is among the most reduced independent multicellular organisms in biology. Pollen is comprised largely of a vegetative cell that forms a pollen tube, which conveys the non-motile sperm cells that it contains into the female gametophyte. The male germline arises from an eccentric division of the post-meiotic haploid microspore that cleaves a relatively small lenticular generative cell from its much larger brother vegetative cell. This sessile generative cell migrates into the vegetative pollen cell and is the founding cell of the male germ lineage. Ultimately the generative cell forms two sperm cells—either in the pollen grain or pollen tube depending on the plant—that constitute the male gametes of flowering plants. Remarkably, both of the male gametes are required in the process of double fertilization. Fusion of one sperm cell with the egg cell results in an embryo—which forms the next generation; whereas fusion of the other sperm cell with the central cell initiates the endosperm—a tissue that is typically a nutritive lineage for the embryo and contributes to its embryonic development. The endosperm and double fertilization are sufficiently unique that they are often used as defining features of angiosperms.

The male gamete has traditionally been the less understood partner in flowering plant reproduction. Although the first realization that flowering plants displayed sexuality began with the work of Camerarius (Zarsky and Tupy, 1995), the realization that pollen grains formed tubes that sought out and entered the ovule began with the work of Giovanni Battista Amici, who proposed that the tube harbored a fertilizing essence that stimulated seed production (Amici, 1824). Wilhelm Hofmeister would later recognize the presence of nuclei in the pollen tubes and document the behavior of their nuclear contents harbored inside pollen (Hofmeister, 1849). Eduard Strasburger, approximately 35 years later published the first details on flowering plant fertilization in which he described nuclear interactions occurring between the male and female gametes constituting karyogamy (Strasburger, 1884). Interestingly, he initially misidentified the conspicuous tube nucleus as the stimulus for the development of the egg cell to form the embryo, but this was rapidly corrected when he observed that the nuclei of sperm were fusing with the egg nucleus. The significance of the second sperm cell was recognized when its nucleus was noted fusing with the polar nuclei of the central cell and initiating the development of the endosperm at the end of the last century, a defining event of double fertilization (Nawaschin, 1898).

The inconspicuous nature of the male generative cell and their subsequent pair of male gametes has led to an underestimation of their importance—which to some extent continues. A century later, some beginning biology texts did not recognize flowering plant sperm as cells, but only bare nuclei—an observation that was mistakenly cited as an important difference from animals. In the early days of molecular biology, these diminutive, non-motile sperm cells were surmised to be completely dependent on the pollen grain/tube nutritionally, and were consequently thought to be dependent on the pollen vegetative cell for all transcription, translation and gene expression (Mascarenhas, 1990).

Recognition that the generative and sperm cells were largely transcriptionally and translationally independent was first shown directly in 1993 (Zhang et al., 1993). It is now clear that flowering plant sperm cells have their own unique patterns of transcription (Gou et al., 2001; Engel et al., 2003), their own unique promoters (Xu et al., 1999; Engel et al., 2005), cell cycle control factors (Borg et al., 2011; Twell, 2011), and silencing elements (Haerizadeh et al., 2006). Furthermore, sperm cell-expressed genes may even control early embryogenetic effects. The zygotically-expressed paternal transcript of *SSP* (*SHORT SUSPENSOR*), as an activator of YODA, was recognized to initiate an asymmetric pattern of cell division of the zygote, which forms a strongly asymmetric and polarized two-celled proembryo that contains a small apical cell at the tip and a larger basal cell (Bayer et al., 2009). This represents the initial deciding point in which the fate of the embryo proper is separated from that of the larger suspensor, establishing the suspensor as a terminal lineage. The male gamete can thus influence gene expression from the first cell division of the next sporophyte generation.

In contrast, the importance of pollen genes reaches its peak during pollen tube competition with other tubes during which their behavioral priority is to preemptively deliver their contained male gametes into a small receptive region of the female gametophyte, targeting the female gametes. The sperm cells meanwhile must be synchronized with the female gametes with respect to cell cycle and receptivity. Double fertilization depends on participation of all gametes, and the fusion of each of the two male gametes with their respective female counterpart. Although the successful competition of the pollen tube is critical to determining the eligibility of male gametes to participate in fertilization, it is only the sperm cell that participates in the subsequent transmission of its paternal genes into the next generation through fertilization. Male gametophytic cooperation results in multiple well-choreographed successes of the two contrasting transcriptomes of the sperm and pollen cells in order to achieve passage into succeeding generations. The male strategy of over-production of pollen at lower energetic cost than egg cells is a strategy of reproduction that is highly conserved between plants and animals (Richards, 1997). This lower investment cost and more abundant production of pollen allows greater variability in the male gametophyte in competing for the ability to fuse with the egg cell and the polar nuclei, but also places high demands on the quality of the gametes and their interactions with the female gametes to assure sustained intergenerational success over time.

## Initiation and origin of the male germ lineage in flowering plants appears to be entirely post-meiotic

Unlike animals which have predestined cells functioning as the germ cell lineage, plant cells appear not to possess a standing population of germ cells during their somatic phase and the emergence of the germ lineage appears to be entirely positionally determined. The diplohaplontic nature of plants maintains obligate passage through single cells at both meiosis and syngamy, alternating with multicellular sporophytic and gametophytic generations. Male gamete formation during normal sexual reproduction in flowering plants occurs through a microsporocyte that undergoes meiosis to form four microspores. When the microspore divides, the generative cell is formed through cell wall formation that partitions the cell in a typically convex pattern, initiating an eccentrically positioned cell plate from the center of the mitotic axis and expanding centrifugally. The formation of the generative cell partitions the pollen into a small lenticular generative cell that occupies approximately 1/20 of the volume of the pollen and a much larger pollen vegetative cell (Russell et al., 1996; Russell and Strout, 2005). The generative cell may further partition cytoplasmic regions that become isolated from the nucleus during development and continues to become smaller during development.

The outer wall of the generative cell originates directly from the microspore intine, whereas the inner wall forms from an interior cellular partitioning of the microspore. This dividing cell wall is somewhat unusual in composition, as it is rich in callose (a β1→3 glucan), which is removed from this region during later maturation. The generative cell migrates into the interior of the vegetative cell through a unique separation mechanism that is correlated with the disappearance of callose labeling on the newly formed crosswall, and an intensification of labeling in the area of separation (Russell et al., 1996). Upon completion of separation of the generative cell from the intine, the generative cell typically polarizes and occupies a unique “cell-within-a-cell” configuration, which precedes the formation of the sperm cells. The generative cell becomes physically associated with the vegetative nucleus, establishing the “male germ unit.” The generative cell and later sperm cells generate cytoplasmically-derived vesicles that appear to reduce their cellular volume throughout development (Yu and Russell, 1992). At maturity, sperm cells may occupy far less than 1% of the volume of the pollen and are among the smallest cells in many flowering plants (Russell and Strout, 2005).

Asymmetry in the volume of the descendent cells appears to be required for the establishment of the male germ lineage. Equational divisions of the microspore giving rise to equal-sized cells result in the formation of two vegetative cells and no reproductive cells (Eady et al., 1995). Interestingly, the *sidecar* mutant can result in two equal-sized cells, initially retaining vegetative identity, but when one of these cells undergoes an asymmetric division, it forms a generative cell that divides to form two apparently completely normal sperm cells (Chen and McCormick, 1996). Dissimilar cell volumes presumably trigger the key transcription factors and activate the developmental program of the male germ lineage (Oh et al., 2011). Reactivation of the cell cycle in the generative cell appears to license the single mitotic division required to form the two sperm cells (Brownfield et al., 2009), whereas further cell cycle progression in the vegetative cell continues to be inhibited.

## Maturation in the germline entails novel structural, physiological and morphogenetic features

The sperm cell surface does not have a traditional cell wall, which would impede fusion, but instead consists of a “periplasm” (McConchie et al., 1987), the nature of which appears to be similar to that of a brush-border. Freeze-substitution preparations have revealed this periplasmic region is characterized by the presence of insoluble polysaccharides, but these do not form discernible fibers, which confirms the absence of a traditional cell wall surrounding the sperm cells (Russell and Cass, 1981). Experiments using living tobacco pollen tubes at generative cell division revealed that newly-formed sperm cells could inadvertently fuse with one another; however, soon after division, the surface of the sperm cells had matured sufficiently that they no longer were able to fuse spontaneously on contact (Tian and Russell, 1998). Addition of a dilute solution of cellulose and pectinase could remove this inhibition, suggesting that multiple barriers to spontaneous fusion may exist. It is possible that carbohydrate moieties on the surface of the sperm cells may even assist in nullifying charge differentials on the surface of the gametes, thus contributing to overcoming the natural repulsion of negatively-charged membrane phospholipids during later fusion (Russell, 1992).

## Cellular condition of pollen, cell cycle positioning of gametes, and gametic cell communication

**Figure 1:**
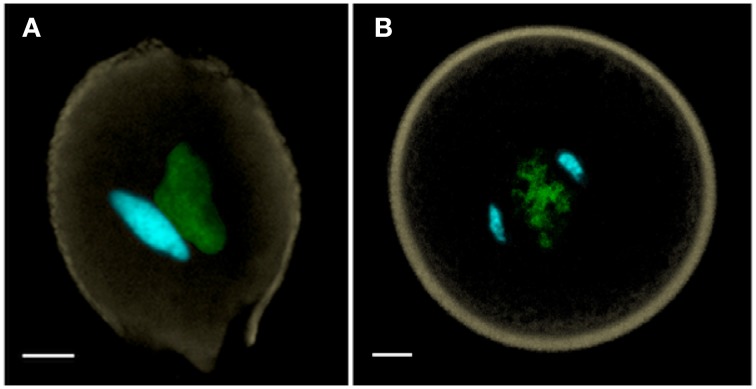
**(A)** Bicellular pollen is exemplified by *Nicotiana benthamiana*, and **(B)** tricellular pollen by *Oryza sativa* (rice). Both of these anthesis pollen grains are labeled with DAPI, captured as a MIP using confocal laser scanning microscopy, and manually-segmented to portray generative and sperm nuclei in cyan, vegetative nucleus in green and pollen wall autofluorescence in beige. Scale bars = 5 μm.

Flowering plant pollen can be released at anthesis in two alternative conditions—one in which pollen is bicellular containing a generative cell—as in ~70% of angiosperms (Figure 1A), or one in which pollen is already tricellular, containing two sperm cells at anthesis, as in the remaining ~30% of angiosperms (Figure 1B) (Brewbaker, 1967). The precocious formation of sperm cells prior to anthesis in tricellular pollen constitutes a heterochronic shift that is generally regarded as apomorphic (Williams et al., 2014). Although there are some species where anthers may even bear both bicellular and tricellular pollen within the same anther, these are rare. The cellular condition of pollen appears to be in evolutionary flux with abundant transitory examples of conversion and reversion of pollen cell types (Williams et al., 2014).

The majority of animals are known to fuse with the gametes in G1 (prior to S-phase in the cell cycle), but angiosperms may fuse in either G1 or G2 phase (Friedman, 1999). While gametic fusion in both G1 and G2 phases occur, no examples of fusion in S-phase are noted. Based on plants studied to date, tricellular pollen may be disseminated with gametes at G1, S or G2 phase, and bicellular pollen is disseminated with generative cells at G1 or G2 (Friedman, 1999). Defects in the control of the cell cycle are well-documented sources of cell identity defects and missed cell cycle cues are an important source of defective gamete behavior, many of which are informative with respect to the control of gamete maturation (Durbarry et al., 2005; Brownfield et al., 2009; Borg et al., 2011, 2014). Cell cycle synchrony appears to be required at the time of gamete fusion. Thus, missed cues can occur at multiple time points in the maturation of the gametes, and may occur late in the life of the gamete, as well. Tobacco sperm cells, which appear to fuse at G2, exemplify very late maturing gametes, as the sperm cells are discharged into the synergid in the G1 condition and fuse in G2. During the protracted time that sperm cells were observed in the receptive synergid, nuclear DNA quantity increased in both male and female gametes until they synchronized at G2-phase, when fusion would occur (Tian et al., 2005). Female gametes in unpollinated flowers would eventually enter G2 phase, but would be delayed by nearly a day and a half without pollination.

Some flowering plants appear to require new gene expression in order for pollen tubes to be able to detect female gametophyte signals and discharge their gametes, particularly with respect to *in vitro*-grown pollen. In Arabidopsis, for example, pollen tube elongation within the style appears to be required to activate a number of genes necessary to control tube guidance and modulate reception in the female gametophyte (Higashiyama and Hamamura, 2008; Palanivelu and Johnson, 2010). As many plants require more than 6 h of pollen tube passage to reach the female gametophyte, it would not be surprising that late expressional changes may occur in the male gamete, especially in bicellular species. A stark contrast to this is rice (*Oryza sativa*), in which fertilization may be effected in <30 min. Comparisons of elongating rice pollen tubes with pollen grains reveal that the most major conspicuous change in rice pollen tubes is the intensification of metabolic response in secretory pathways with few other detectible changes in gene expression (Dai et al., 2006; Wei et al., 2010).

## Male germ lineage transcripts and products reflect rich, complex and dynamic gene expression

That gene expression in the male germ lineage would include a rich assemblage of transcriptional and translational products was evident from the early-1990s, when ESTs of sperm and generative cell cDNA libraries were first examined and sequenced (Zhang et al., 1993; Blomstedt et al., 1996). Among the earliest discovered novel proteins were those involved in chromatin changes in the male germ lineage, which included an unusually rich complement in substitution histones H2A, H2B, and H3 that displayed comparatively low homology in the highly conserved histone gene family (Ueda and Tanaka, 1995a,b). The lily generative cell lineage that Ueda and Tanaka studied had long been observed to have distinctive chromatin configurations in the nuclei of generative and sperm cells and the large size of the generative nuclei were attractive, particularly given the relative insensitivity of molecular methods at that time. Their studies revealed novel chromatin-related genes that encoded a number of variant histones, which were later observed in Arabidopsis (Okada et al., 2005b), soybean (Haerizadeh et al., 2009), rice and a number of other model angiosperms (Singh and Bhalla, 2007). These epigenetic factors continue to be a major theme in modern work as well.

Promoter analysis has revealed that there are male germline-selective promoters that are activated in the generative and sperm cells (Xu et al., 1999; Okada et al., 2005a). Whereas most promoters are positively controlled, there is also evidence for a complex silencing element that controls male germline expression through a repressor that is expressed in all but male germ cells (Haerizadeh et al., 2006). In the latter case, without the repressor protein, male germline genes are constitutively expressed. Conservation of DNA repair genes is suggested by the characterization of a lily generative cell homologe to the human excision repair gene ERCC1 (Xu et al., 1998). This supports that conservation of DNA repair enzymes may extend among distant groups of eukaryotes (Tuteja et al., 2001). The presence of a diversity of DNA repair enzymes in the male germ lineage is supported by evidence from in a number of expression libraries (Okada et al., 2006; Borges et al., 2008; Abiko et al., 2013b).

Activation of ubiquitin pathways contributes to increased rates of protein turnover and also represents evidence of protein dynamism. The ubiquitin pathway involves activating E1 enzymes, ubiquitin-conjugating E2 enzymes, and ligating E3 enzymes that link ubiquitin to proteins, thus targeting them for degradation in the proteosome pathway. Highly transcribed members of the ubiquitin pathway are common in transcriptomes of male germ-related lineages and may even be differentially enhanced in different male germ cells (Singh et al., 2002).

## Male germline cells and pollen display unique profiles, distinct gene complements

Transcriptomic analyses of the developing cells of the male gametophyte of Arabidopsis using microarrays revealed complex patterns of gene regulation throughout pollen maturation, with a sharply decreasing number of active genes from the uninucleate microspore, to generative cell initiation in bicellular pollen, with still fewer genes expressed in tricellular pollen and post-anthesis pollen (Honys and Twell, 2004). The transcriptome of mature, anthesis pollen revealed a functional complement of genes highly upregulated in cell wall metabolism, cytoskeleton and cell signaling, but otherwise reflected a cell with a short remaining lifespan and an overall unsustainable metabolism—a transcriptomic composition reflective of its limited behavioral possibilities (Becker et al., 2003; Honys and Twell, 2003; Pina et al., 2005). This divergent and restricted expressional profile appears to be conserved in the anthesis pollen of multiple species of angiosperms examined to date, including soybean (Haerizadeh et al., 2009), rice (Wei et al., 2010), and tobacco (Hafidh et al., 2012).

As with all molecular and biochemical assays, sufficient high quality male gametes have to be available, combined with adequate detection sensitivity, to characterize the gametes. Two major protocols have emerged for the isolation of male gametes: (1) differential centrifugation, typically requiring the collection of cells from a continuous Percoll or discontinuous sucrose density gradient (Russell, 1991), and (2) fluorescence-activated cell sorting (FACS), using a sperm-selective promoter to drive the expression of a GFP reporter in order to label the targeted cells for isolation (Engel et al., 2003, 2005; Borges et al., 2008, 2012b). Both techniques produce samples that are sufficiently pure to separate enriched transcripts from the male germline cells with sufficient collections. Needless to say, vastly different quantities of pollen are available in wind-pollinated plants than in insect-, bird- or self-pollinated plants (Richards, 1997).

Transcriptomes of male gametes have clearly illustrated the divergent nature of gene expression in the male germ lineage compared to that of the vegetative pollen. Estimates of the number of genes present in the sperm, pollen and seedlings of Arabidopsis have yielded microarray presence calls of 27% for sperm cells (corresponding to 5829 genes), 33% for pollen (corresponding to 7177 genes), and 64% for seedlings (corresponding to 14,464 genes) using the MAS5 algorithm and gene counts normalized to the Arabidopsis genome (Borges et al., 2008). Among transcriptional themes encoded by the sperm cells are DNA repair, ubiquitination, and cell cycle progression, which are common emerging themes. Gene expression estimates for rice, based on normalized triplicate microarray results using MAS5 unanimous presence calls yielded 10,732 sperm genes, 8101 pollen genes, and 15,449 seedling genes (Russell et al., 2012). The most highly represented functional categories in rice sperm cells involved metabolism, transcription and cell signaling. Additional functional categories up-regulated in sperm cells, as compared with other tissues, include transcription factors, cell signaling, protein modification, cellular identity and receptor-like molecules; these categories may each include some key players in functions unique to sperm cells.

The most sensitive and presumably accurate estimates for transcribed genes of the gametes and pollen are those available from RNA-Seq results (Anderson et al., 2013). Total reads using rice, indicated expression of up to ~25,000 genes in the sperm cell, ~29,000 genes in the pollen vegetative cell and ~27,000 genes in the egg cell, which were far higher than original microarray results. When gene counts were limited to sequences found in all three replicates of each cell, there were 16,985 genes detected in the sperm cell, 18,611 genes in the pollen vegetative cell and 21,172 genes in the egg cell (Anderson et al., 2013). Clearly the original expressional assays underestimated the breadth and depth of these cellular profiles, but they do not begin to answer the question of how many of these are translated and how many occupy essential roles. Based on microarray results, there is clearly a broad group of genes expressed in all of the cells that may reflect a “housekeeping” role, providing core metabolic functions.

**Figure 2:**
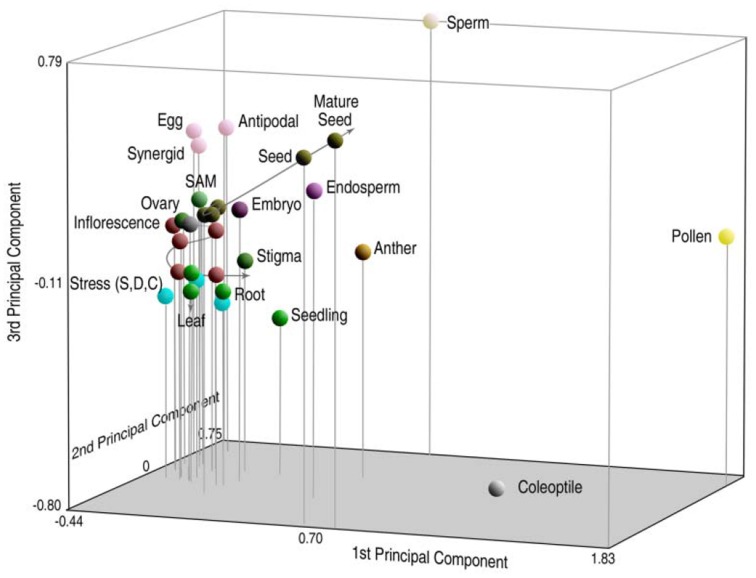
**Principal components analysis (PCA) of probe set signal intensity on the rice transcriptome, reflecting gene expression patterns in 31 different tissues of rice**. Relative distances of sperm profiles from those of other cell and tissue types, including pollen, are indicative of highly divergent patterns of overall gene expression in the male germ lineage. Reproduced with permission of J.C. Wiley Press (Russell et al., 2008, 2012).

To compare the relationship of genes transcribed in a gallery of different tissues, principal component analysis (PCA) was used to portray *n*-dimensional data sets on smaller 3D axes so that the components of greatest variation could be compared (Russell et al., 2012). Relative distances on the three-dimensional graph are proportionate to their degree of relatedness in *n*-dimensions. In Figure 2, rice sperm cells are compared with microarray expressional data from 31 different tissues, including sperm and pollen (representing the male gametophyte), 26 different vegetative and reproductive tissues of the sporophyte (representing different sporophytic phases of the life cycle, including different tissue conditions, organs, developmental conditions, and environmental responses) and three different tissues representing the female gametophyte. Interestingly, the high degree of divergence in expression between sperm and the pollen vegetative cell place them as anchor values on multiple axes and place them at a significant distance from sporophytic expression. These differences between tissues remain high, even when compared with the anther, which includes pollen and sperm as a subset (Russell et al., 2012). Overall expression also differs between the male germline and differentiated sporophytic tissues in other analyses as well (Borges et al., 2008; Abiko et al., 2013b).

The RNA-Seq data revealed expression profiles reflecting an upregulation of genes involved in chromatin conformation, indicating an unexpected degree of chromatin activation in the sperm cells. The transcriptomes of the egg and sperm reveal major differences in gene expression that will presumably be altered within the zygote. These differences represent the native state for parent-of-origin gene expression and will be a baseline for further studies of the zygote during fertilization and early embryogenesis. Particularly pathways affecting epigenesis, methylation, hormonal control, cell cycle and specific gametic functions were examined in anticipation of their potential contributions to early zygotic and seed development (Anderson et al., 2013). Notably, three-quarters of the genes were differentially expressed between cell types.

## Proteomic profiles of egg and sperm lineages have many common elements, few specifically divergent proteins

Proteomic data on isolated generative cells and male gametes have been obtained for a limited number of species. In Arabidopsis, translation of a number of gene products have been confirmed, but not all transcripts are translated into proteins within the male germ lineage (Bayer et al., 2009). Lily was selected as a model bicellular pollen species in which protein expression profiles of GCs and SCs were examined by 2D-DIGE, displaying about 2500 protein spots with 226 displaying significant changes in expression; 124 were upregulated during SC development whereas 102 were downregulated (Zhao et al., 2013). Of the annotated proteins detected, 71% were involved in six main functional groups—metabolism, cell cycle, signaling, ubiquitin/proteasome pathway, chromatin remodeling, and stress response (Zhao et al., 2013).

In rice, 2138 proteins were detected in the egg cell and 2179 proteins in the sperm cells. A total of 102 proteins were preferentially expressed in the egg cell and 77 proteins were preferentially expressed in the sperm cells (Abiko et al., 2013a). Proteins selectively enriched in the egg cell proteome appeared to reflect a functionally diverse collection of polypeptides. Proteins enriched in the sperm proteome appeared to reflect narrower motifs and were more centered to fusion-related functions (Boavida et al., 2013). Such selectively expressed transcripts encoded proteins including such motifs as protein modification, lipid-related proteins, and potential cell surface modifying proteins (Abiko et al., 2013a).

## Transposable elements in pollen and male germline

An enigmatic feature of the male germ transcriptome is the frequent occurrence of sequences that encode transposable elements (TEs), which may be present in remarkably differing quantities in different plants and different environmental conditions. The vegetative cell is known to be the site of considerable TE activity, which is prevalent enough that it has been directly observed using transposon displays in pollen (Turcich and Mascarenhas, 1994). LTR retrotransposons, which are particularly highly activated in the pollen, are believed to be strongly suppressed in the male germline (Slotkin et al., 2009). Sperm cells are regarded as displaying a high degree of DNA-level methylation, which is believed to play a central factor in suppression of TEs and follows three nucleotide motifs in flowering plants: CG, CHG, and CHH (where H may equal A, C, or T). Sequences to be silenced are typically encoded by RNA-directed DNA methylation through the action of small RNAs whereas CG and CHG motifs remain highly methylated in the male germ lineage, suppressing the activity of retrotransposons. The symmetrical nature of CG and CHG motifs confers similar methylation patterns in each DNA strand, resulting in similar epigenetic markings in their progeny, whereas the activation of DNA glycosylation, which demethylates DNA, is evident in the vegetative cell and activates DNA transposons at imprinted loci (Borges et al., 2012a). CHH motifs in microspore and sperm cells are believed to undergo a dramatic decrease in methylation in the male germ lineage. Although demethylation could compromise the repression of DNA transposons, methylation is not restored until the zygotic stage (Calarco et al., 2012). Upon fertilization, methylation is believed to be reactivated in the zygote by siRNA-based silencing elements from the pollen and the endosperm, which appear to control site-specific methylation. Chromatin-related changes in methylation are also a key consequence of combining the gametes, each of which have their own signature molecules (Slotkin et al., 2009). Activation of CHROMOMETHYLASE 3 (CMT3) appears to alter methylation at both CG and CHH sites, whereas the male germ lineage is relieved from TE suppression through the activity of (DRM2); this in turn is believed to release TEs from suppression by the histone methyltransferases KRYPTONITE (KYP/SUVH4) and SUVH5/6 (Calarco and Martienssen, 2011).

In the male gametes of Arabidopsis (Borges et al., 2008), lily (Okada et al., 2006), and *Plumbago* (Gou et al., 2009) relatively few TEs are transcribed, but in grasses such as maize (Engel et al., 2003) and rice (Russell et al., 2012), the high genomic content of TEs appears to be reflected in abundant transcripts. Sperm cell ESTs collected from maize pollen at anthesis had approximately 9.46% annotated retrotransposons according to GenBank accessions, whereas DNA transposons represented only 0.06% of the transcripts. Although retrotransposon content may be proportionate to TE content in the maize genome, DNA transposons were scarce, suggesting that a combination of DNA-level methylation and chromatin modification may dominate the repression motifs in the epigenome of maize sperm, as is known to occur elsewhere (Borges and Martienssen, 2013). Further suppression of TEs through such short RNA species as siRNAs, are believed to convey a high degree of precision to the process of silencing (Creasey et al., 2014). Consistent with the conservative gender-based behavior seen in other eukaryotes, TE transcription does not seem prevalent in egg cells (Anderson et al., 2013). Female gametophytes of Arabidopsis displayed less than one-fifth the number of retrotransposons (1.69%) and more annotated DNA transposons (1.44%) than in maize (Yang et al., 2006). Differentially fewer female-transcribed TE motifs were also observed in rice (Anderson et al., 2013), suggesting that a generally lower number of TE transcripts are present. Such reduced TE activity may be a consistent feature among female germ lineages. Whether this reflects diminished transcription of TE genes or greater success in suppression of TE transcripts is not immediately evident. In either case, TE abundance is clearly less in the female gametes than in the male gametes (Russell et al., 2012; Anderson et al., 2013).

## Contribution of the sperm cells upon fertilization

**Figure 3:**
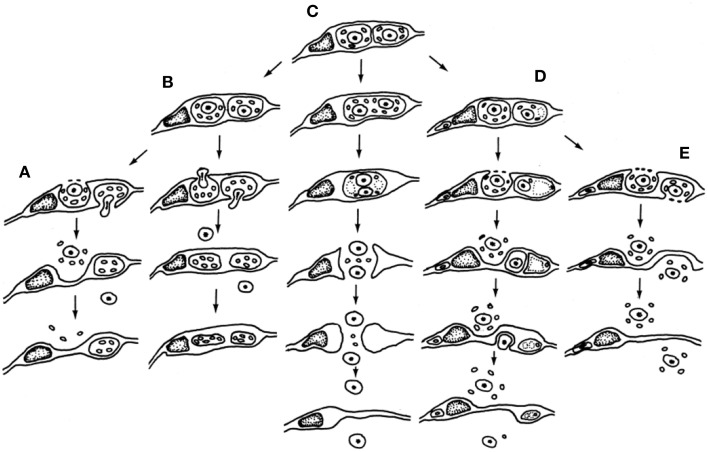
**Participation of male germline cytoplasmic organelles shows incorporation, partial incorporation, or exclusion of heritable cytoplasmic organelles (often restricted to mitochondria because of prior plastid exclusion or elimination from the generative cell)**. Five models have been described and documented using electron microscopy. **(A–D)** Models favoring uniparental maternal inheritance. **(A)** In barley, paternal organelles are excluded from egg but not central cell (Mogensen, 1988); **(B)** In cotton, paternal organelles are excluded from both embryo and endosperm (Jensen, 1964); **(C)** binucleate model of (Wilms, 1981) in spinach; **(D)** in *Populus*, organelles are excluded by extra-cytoplasmic body production (Russell et al., 1990). **(E)** Composite model of biparental cytoplasmic transmission, based on *Petunia* (Van Went, 1970) and *Plumbago* (Russell, 1983). Reproduced with permission of Springer-Verlag (Russell et al., 1990).

The paternal nucleus represents half of the genomic complement of the zygote—thus an equal partner in the formation of the embryo and resulting sporophyte—but the quantity of transcripts encoded and expressed greatly differ, which by definition forms a starting point for gametic parent-of-origin effects (Luo et al., 2014). Size differences between the male and female gametes would nearly ensure a greater quantity of female gamete-expressed transcripts in the zygote, but this input alone does not eliminate potential impacts of a wide variety of epigenetic factors that may alter the number of functional transcripts delivered, expressed or sequestered. Cytoplasmic ratios between male and female gametes may be <1:50 in the zygote and <1:800 between the male gamete and central cell cytoplasm that constitutes the endosperm (Russell, 1987). A relatively low ratio of paternal-to-maternal cytoplasmic volume allows minimal opportunity for the transmission of sperm-delivered heritable organelles and likely this is a common event during fertilization. Such interactions may be entirely eliminated in species such as barley, in which the cytoplasm is excluded and the sperm cell cytoplasm appears to remain amid degenerating pollen and synergid cytoplasm outside of the egg cell (Mogensen, 1988). Other species may transmit varying amounts of paternal cytoplasm (Russell et al., 1990). Figure 3 displays five different patterns of male cytoplasmic transmission that have been reported in the literature to date, and which lead to a predicted relaxed control of heritable organelle contributions (Birky, 1983). Yet, paternal organelles occur in the sperm cells of tobacco, that are detected within the zygote and early embryo genetically (Yu et al., 1992, 1994) and there is molecular evidence of transmission of nuclear transcripts, as well (Ning et al., 2006). Paternal transcripts of *SSP* (*Short Suspensor*) are known to be transmitted into the zygote of Arabidopsis, translated, and their products expressed in the fertilized egg cell. As the SSP protein activates expression of YODA, this male contributed protein sequence establishes the asymmetry of the two-celled proembryo (Bayer et al., 2009). The diversity and distinctiveness of paternal transcripts in the sperm cells seems to suggest a role in shaping parental elements of the transcriptome of the zygote early in development (Russell et al., 2012). That the dimorphic sperm cells of *Plumbago zeylanica* (Russell, 1984) have different preferential fates during fertilization (Russell, 1985) further suggests that their differentially expressed transcriptional complements may target female cells and contribute to their different fates (Gou et al., 2009).

The paternal genome of animals is often silenced prior to the maternal to zygotic transition (MZT), thus the onset of zygotic expression in animals coincides with a suppression of messages from the egg cell and the onset of expression from both sets of chromosomes (Baroux et al., 2008). In plants, however, maternal and paternal chromosomes appear to be equal contributors from the earliest stages of embryogenesis (Nodine and Bartel, 2012). The endosperm, which typically receives two copies of the maternal genome, often displays strong evidence of maternal imprinting in the endosperm, which coincides with rapid demethylation of DNA, and thus the activation of the endosperm lineage (Bauer and Fischer, 2011). However, changes in gene activation in the zygote proceed methodically, involving removal of some histone variants, such as MGH3 substitution histones in the embryo lineage (Ingouff et al., 2010). In rice, only a handful of genes in the embryo displayed imprinting and in all three cases these were maternal. In contrast, the endosperm had just over 2% of its genes discernibly imprinted. In the endosperm, however, unlike the embryos, the maternal-to-paternal imprinted genes neared a 2:1 ratio of contributed genomes of the two polar nuclei relative to the sperm nucleus (Luo et al., 2011).

## Male expression and evolutionary selection

The degree to which male germ cells undergo effective evolutionary selection can be judged by the degree to which altered nucleotides in their genes are replaced with nucleotides encoding the same amino acid sequence, indicative of purifying selection, as opposed to random replacement. Haploid regimes, which are by definition not masked by dominant genes, are particularly adaptable to selection. For example, mosses displaying purifying selection may be very effectively selected in strongly conserved phenotypes with highly expressed protein-coding regions (Szövényi et al., 2014). In pollen vegetative cells and pollen tubes, 6–11% of important genes display purifying selection with purifying selection in pollen far exceeding that in seedlings (Arunkumar et al., 2013). Adaptations that favor pollen tube competition appear to be strongly selected in the population. In contrast, genes expressed in sperm cells display fewer sites that are under strong purifying selection than either seedlings or pollen (Arunkumar et al., 2013). Although genes expressed in gametes and synergids show high rates of protein evolution, a greater proportion of adaptive amino acid substitutions are the result of increased levels of purifying selection in pollen and pollen tube-specific genes. Prezygotic sexual selection involving interactions such as pollen tube competition may therefore be more successful than gametes at positive trait selection (Gossmann et al., 2013). Sperm-expressed mutants involved in pollen tube guidance, however, such as *hap2* may display unusually strong positive selection, as gametic interactions may result in increasingly complex patterns of communication designed to optimize success in later seed production (Beale and Johnson, 2013).

Among model systems for reproduction, *P. zeylanica* is one of the most remarkable because it has dimorphic sperm cells in which the fusion fate of the sperm is known from inception. The sperm cell that is associated with the vegetative nucleus (S*_vn_*) is known to preferentially fuse with the central cell forming the endosperm, whereas the other sperm cell (S*_ua_*) preferentially fuses with the egg cell to produce the embryo (Russell, 1985). In order to characterize the sperm transcriptome of these two cell types in the absence of working transformation system (Wei et al., 2006), it was necessary to collect sperm cells using a micromanipulator (Zhang et al., 1998). Using collections of 12,000 sperm cells of each morphotype, representative sperm cell cDNA libraries and custom microarrays were constructed, ESTs characterized, and each sperm cell's functional profiles were compared. Surprisingly, the functional profile of the sperm cells appeared to coincide closely with their putative fusion product (Gou et al., 2009). Thus, the functional profile of the S*_vn_* appeared similar to an expected endosperm-enriched profile, whereas that of the S*_ua_* appeared more similar to an embryo profile. This appears to represent an instance where the precocious development of the embryo may be accelerated by providing targeted paternal genes to be activated upon double fertilization. With modern increases in the molecular sensitivity of characterization techniques and use of greater resolution techniques such as RNA-Seq, the accuracy of this prediction could be examined and potentially tested, providing essentially transcriptomic coverage during early embryogenesis to test the role of male gamete transcriptomes in early post-fertilization development.

### Conflict of interest statement

The authors declare that the research was conducted in the absence of any commercial or financial relationships that could be construed as a potential conflict of interest.
